# Pharmacist-Driven Implementation of Fast Identification and Antimicrobial Susceptibility Testing Improves Outcomes for Patients with Gram-Negative Bacteremia and Candidemia

**DOI:** 10.1128/AAC.00578-20

**Published:** 2020-08-20

**Authors:** Sahil Sheth, Michael Miller, Angela Beth Prouse, Scott Baker

**Affiliations:** a Department of Pharmacy Services, Anne Arundel Medical Center, Annapolis, Maryland, USA; b Department of Pharmacy, Peninsula Regional Medical Center, Salisbury, Maryland, USA; c Department of Microbiology, Peninsula Regional Medical Center, Salisbury, Maryland, USA

**Keywords:** bacteremia, outcomes, pharmacist, AXDX, AST, fast identification, patient outcomes

## Abstract

Bloodstream infections (BSI) are associated with increased morbidity and mortality, especially when caused by Gram-negative or fungal pathogens. The objective of this study was to assess the impact of fast identification-antimicrobial susceptibility testing (ID/AST) with the Accelerate Pheno system (AXDX) from May 2018 to December 2018 on antibiotic therapy and patient outcomes. A pre-post quasiexperimental study of 200 patients (100 pre-AXDX implementation and 100 post-AXDX implementation) was conducted. The primary endpoints measured were time to first antibiotic intervention, time to most targeted antibiotic therapy, and 14-day hospital mortality. Secondary endpoints included hospital and intensive care unit (ICU) length of stay (LOS), antibiotic intensity score at 96 h, and 30-day readmission rates. Of 100 patients with Gram-negative bacteremia or candidemia in each cohort, 84 in the preimplementation group and 89 in the AXDX group met all inclusion criteria. The AXDX group had a decreased time to first antibiotic intervention (26.3 versus 8.0, *P* = 0.003), hours to most targeted therapy (14.4 versus 9, *P* = 0.03), hospital LOS (6 versus 8, *P* = 0.002), and average antibiotic intensity score at 96 h (16 versus 12, *P* = 0.002). Both groups had a comparable 14-day mortality (0% versus 3.6%, *P* = 0.11). In this analysis of patients with Gram-negative bacteremia or candidemia, fast ID/AST implementation was associated with decreased hospital LOS, decreased use of broad-spectrum antibiotics, shortened time to targeted therapy, and an improved utilization of antibiotics within the first 96 h of therapy.

## Abstract (precis)

Bloodstream infections (BSI) are associated with increased morbidity and mortality, especially when caused by Gram-negative or fungal pathogens. The objective of this study was to assess the impact of fast identification-antimicrobial susceptibility testing (ID/AST) with the Accelerate Pheno system (AXDX) from May 2018 to December 2018 on antibiotic therapy and patient outcomes. A pre-post quasiexperimental study of 200 patients (100 pre-AXDX implementation and 100 post-AXDX implementation) was conducted.

## INTRODUCTION

Bloodstream infections (BSI) are associated with increased morbidity and mortality, especially when caused by Gram-negative or fungal pathogens (1). Pathogen identification (ID) and antimicrobial susceptibility testing (AST) are essential tools for appropriate treatment of BSI. Early and effective antimicrobial administration is essential to improve patient outcomes and overall survival (2). Every hour of delay in initiating appropriate antimicrobial therapy in patients with sepsis decreases survival by approximately 8% (2–4). While multiple fast ID systems can identify pathogens within 2 h, most require conventional culture methods for final AST (5). This prevents clinicians from de-escalating therapy for Gram-negative infections due to a variety of resistance mechanisms and a potential of intrinsic multidrug resistance that is not captured by resistance gene testing. Two main technological advances enable early pathogen-directed therapeutic interventions. These include implementation of molecular methods to identify bacteria and yeast present in positive blood cultures along with select antibiotic resistance markers. The second is fast phenotypic susceptibility testing performed directly from the positive blood culture bottle, which provides MIC-level antimicrobial susceptibility data. In comparison to conventional culture methods, these technological advances can optimize microbiology workflows, decrease time to result, and offer clinicians the potential to improve time to antibiotic tailoring (6). Studies of rapid PCR-based organism identification and antimicrobial resistance markers have shown improved outcomes such as shortened time to targeted therapy, reduced time to antimicrobial de-escalation, decreased costs, and reduced patient hospital length of stay (LOS) (7–12). However, these evaluations have been limited to mostly Gram-positive (GP) BSI, and two rapid blood culture diagnostic methodologies have not been compared. Moreover, a comparison of patient outcomes between rapid molecular ID and fast ID and phenotypic AST has yet to be published (7–9, 11).

The Accelerate Pheno system and the Accelerate PhenoTest BC kit (AXDX) is a novel fully automated and FDA-cleared solution using fluorescence *in situ* hybridization-based ID and phenotypic AST direct from positive blood cultures. The system produces ID results in 2 h and AST results in an additional 5 h for a total turnaround time of 7 h (13). Gram-negative pathogens identified by AXDX are Acinetobacter baumannii, *Citrobacter* species, *Enterobacter* species, Escherichia coli, *Klebsiella* species, *Proteus* species, Pseudomonas aeruginosa, and Serratia marcescens. Fungal pathogens identified by AXDX are Candida albicans and Candida glabrata. The impact of this technology on antimicrobial stewardship and clinical outcomes for patients with Gram-negative bacteremia compared to those with rapid genotypic testing remains unclear. In this study, we investigated the clinical utility of fast ID and AST via AXDX on time to therapy interventions, antimicrobial utilization, and overall patient outcomes (mortality, length of stay, and readmission rates) compared to those with VERIGENE genotypic testing.

## RESULTS

### Patients.

**FIG 1:**
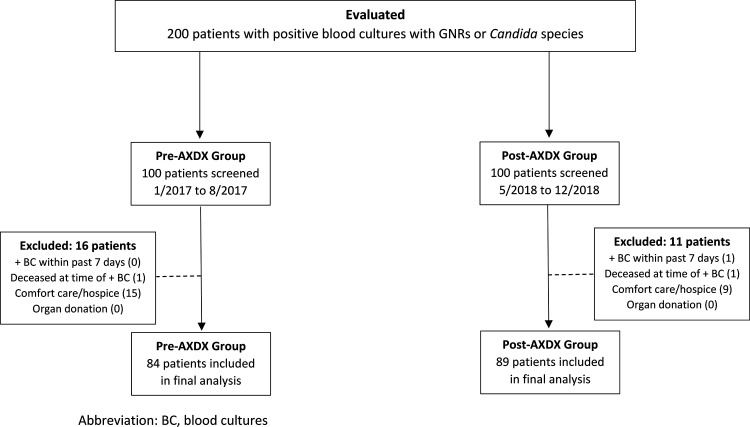
Flowchart of study patients.

A total of 200 patients with positive blood cultures with Gram-negative rods (GNRs) or *Candida* species and hospital admission for greater than 24 h were identified during both study periods. A total of 84 patients in the pre-AXDX implementation group and 89 in the post-AXDX implementation group were included in the final analysis (Fig. 1). There were no statistical differences between patient age, sex, level of immunosuppression, diagnosis of septic shock, or Charlson comorbidity score between the groups. A higher percentage of patients in the pre-AXDX group were admitted to the intensive care unit (ICU) during hospitalization than in the post-AXDX group (*P* = 0.04) There were no statistical differences between other clinical and demographic characteristics except ICU admission, which was higher in the pre-AXDX implementation group (Table 1).

**TABLE 1:** Baseline patient demographics and clinical conditions

Characteristic	No. (%) or median (IQR)^*a*^		*P* value
	Pre-AXDX group (*n* = 84)	Post-AXDX group (*n* = 89)	
Age (yrs)	71 (60–79)	70 (60–79)	0.88
Female	42 (50)	48 (53.9)	0.60
Immunosuppression	13 (15.5)	19 (21.4)	0.32
Charlson comorbidity score	5 (3.0–7.0)	5 (3.5–8.0)	0.29
Septic shock diagnosis	13 (15.5)	7 (7.9)	0.12
ICU admission	24 (28.6)	13 (14.6)	0.04
Source of infection			0.27
Urine	56 (66.7)	44 (49.4)	
Intraabdominal/biliary	12 (14.3)	20 (22.5)	
Line related	7 (7.9)	6 (6.7)	
Other/unknown	2 (2.2)	11 (12.4)	
ID consulted	24 (28.6)	33 (37.1)	0.23
Prior hospitalization within 90 days	22 (26.2)	28 (31.5)	0.23

a IQR, interquartile range.

### Microbiology.

In the pre-AXDX implementation group, positive blood culture identifications consisted of 62% E. coli, 17% Klebsiella pneumoniae, 7% Proteus mirabilis, 5% P. aeruginosa, and 9% other GNRs (see Table S1 in the supplemental material). In the post-AXDX implementation group, identifications consisted of 46% E. coli, 19% *Klebsiella* species, 7% *Proteus* species, 6% *Enterobacter* species, 4% P. aeruginosa, and 18% other GNRs (Table S1). E. coli was the only pathogen statistically significant between the two study groups (*P* = 0.037). One *Candida* species was isolated in each group. The sensitivity and specificity for AXDX for organism ID was 100% when verified by conventional microbiology methodology.

The most common source of bacteremia was urinary followed by intraabdominal/biliary in both pre-AXDX and post-AXDX implementation groups (Table 1). A urinary source of bacteremia was more common in the pre-AXDX implementation group (66.7% versus 49.4%, respectively; *P* = 0.02).

### Antimicrobial use and stewardship outcomes.

Primary, secondary, and other predefined endpoints of the study are summarized in Table 2. Time to first antibiotic intervention was significantly shorter in the post-AXDX group than in the pre-AXDX implementation group (8 versus 26.3 h, respectively, *P* = 0.003). Median time to targeted therapy was also significantly shorter in the post-AXDX group (9 versus 14.4 h, *P* = 0.03). Median days of broad-spectrum antibiotic use (1 versus 3 days, *P* < 0.0001) and antibiotic intensity score (12 versus 16, *P* = 0.0002) were reduced in the post-AXDX group. All of these endpoints remained statistically significant when restricting the analysis to non-ICU patients, with the exception of time to targeted therapy, which was comparable between groups (median: pre-AXDX, 8 h; post-AXDX, 10 h; *P* = 0.17). The targeted antibiotic regimen most commonly used in patients in pre-AXDX and post-AXDX implementation groups was ceftriaxone monotherapy, which was approximately 55% in each group (see Table S2).

**TABLE 2:** Primary, secondary, and other predefined endpoints^*a*^

Endpoint	Median (IQR) or no. (%)		*P* value
	Pre-AXDX group (*n* = 84)	Post-AXDX group (*n* = 89)	
Time to first antibiotic intervention (h)	26.3 (4.5–43.6)	8 (6.5–11.3)	0.003
Time to most targeted therapy^*a*^ (h)	14.4 (0–49.6)	9.0 (0–18.5)	0.03
14-day mortality	3 (3.6)	0	0.11
Hospital LOS (days)	8 (6–10.75)	6 (4.5–8.5)	0.002
Hospital LOS post-positive BC^*b*^ (days)	6 (4–9)	5 (3–7)	0.01
ICU LOS post-positive BC (days)	3 (2–6.25)	2 (2–2.5)	0.25
Antibiotic intensity score^*c*^	16 (10.5–20)	12 (9–15.5)	0.0002
30-day readmission	7 (8.6)	5 (5.6)	0.44
Broad-spectrum antibiotics (days)	3 (2–3)	1 (0.5–2)	<0.0001

a After positive blood cultures.

b BC, blood cultures.

c Calculated at 96 h of antibiotic therapy.

There was a higher percentage of antimicrobial stewardship interventions in the post-AXDX group than in the pre-AXDX group (40.4% versus 19.0%, respectively; *P* = 0.002). Recommendations were most commonly de-escalation (11.9% versus 33.7%), escalation/initiation (4.8% versus 4.5%), and change/modification (2.4% versus 2.2%) in both study periods.

### Clinical outcomes.

There were no statistically significant differences in 14-day mortality in the post-AXDX group (0% versus 3.6%, *P* = 0.11). There was a statistically significant difference between pre-AXDX and post-AXDX implementation groups in hospital LOS (8 versus 6 days, respectively; *P* = 0.002), and it remained significantly shorter in the post-AXDX (median, 5 days; *P* = 0.02) than in the pre-AXDX group (median, 7 days) when restricting the analysis to only non-ICU patients only. There were no significant differences in ICU LOS or 30-day readmission between the two groups (Table 2).

## DISCUSSION

In a community hospital where infectious diseases specialty services are not available 24 h, 7 days a week, we sought to integrate fast diagnostics in combination with pharmacy-driven antimicrobial stewardship to improve patient outcomes. Our results demonstrate that in a resource-limited community hospital setting, fast ID and AST via AXDX can be used in conjunction with clinical pharmacy services to positively impact patient care. Additionally, due to an observed average hospital LOS reduction of 2 days, potential cost savings can be realized. Cost-effective initiatives are essential for community hospitals, especially in suburban settings, where financial viability is key.

To our knowledge, this is one of only a few studies to evaluate a fast diagnostic test on antimicrobial stewardship and clinical patient outcomes for GNR and *Candida* BSI at a community hospital. Lockwood et al. demonstrated a significant reduction in time to therapy adjustment and hospital costs using matrix-assisted laser desorption ionization–time of flight (MALDI-TOF) mass spectrometry and near real-time pharmacist notification in comparison to conventional ID and AST for Gram-negative bacteremia (10). However, no difference in hospital LOS was observed in their study (10). Our study results are also consistent with others in the literature that have demonstrated benefits of fast diagnostics in reducing time to first antibiotic intervention, time to targeted therapy, hospital LOS, and other clinical outcomes in primarily GP BSI (4–6, 8, 9). Nevertheless, this study contributes new information on the impact of fast diagnostic tests compared to others previously published literature. First, it adds the perspective of utilizing fast ID and AST for GNR or *Candida* BSI as the popularity of using such diagnostic methodologies increases. Additionally, this is the first study to compare fast ID and AST (AXDX) to a standard of care with established fast ID and resistance gene testing (VERIGENE system) followed by conventional AST.

Our findings highlight the collaboration and workflow optimization between pharmacists, providers, and microbiology laboratory personnel. Such meaningful reductions in time to first antibiotic intervention and time to targeted therapy results would not have been possible without the technology as well as the commitment of these stakeholders in the hospital. We observed that providers were more willing to de-escalate empirical antimicrobial therapy after final AST (provided by AXDX) as opposed to after ID and resistance gene results alone, primarily due to the possibility of undetected resistance with genotypic testing. This is similar to that for other institutions that have shown time from Gram stain to ID and AST, time to optimal therapy, time to step-down antimicrobial therapy, and length of stay outcomes through AXDX utilization (14, 15). This earlier de-escalation of antimicrobial therapy in *Enterobacteriaceae* bloodstream infections can significantly help decrease Clostridioides difficile infection rates, as recently reported in literature (16).

This study is not without limitations, which include a single-center design, making it less generalizable to hospitals with dissimilar patient populations. Second, differences in antimicrobial stewardship program involvement need to be addressed when determining the generalizability of these data to other centers. Third, microbiology laboratory staffing during the post-AXDX period to run AXDX on the evening shift was greater than what was available during the pre-AXDX period. This could have resulted in delays for final ID and AST in the pre-AXDX implementation group. In addition, during the post-AXDX period, the on-call infectious diseases/critical care pharmacist was paged if *Pseudomonas*, Acinetobacter, or *Candida* species were isolated, with subsequent adjustment of therapy through provider paging. This service was not available during the pre-AXDX period, which could have resulted in variabilities of antibiotic modifications and patient outcomes. However, all other pharmacy stewardship services remained unchanged between the study periods. It is important to note the different seasonal time frames of both groups, which could account for higher variability of GNRs observed in the post-AXDX group, particularly *Vibrio* and *Salmonella* species. There were minimal *Candida* species isolated in each group, which decreases the applicability of the study findings for those pathogens. There were more patients admitted to the ICU in the pre-AXDX implementation group, which could impact many of the endpoints evaluated in the study. However, when removing ICU patients from the analysis, the majority of associations observed in the study remained statistically significant. Lastly, the study sample size was not powered to detect a difference in 14-day mortality. Despite these limitations, this is the first trial that investigated the clinical utility of fast ID and AST for GNR and *Candida* BSI in a community hospital with existing rapid testing methodology as a conventional comparator and observed the impacts on antimicrobial stewardship and patient outcomes.

In conclusion, fast ID and AST implementation via the AXDX system was associated with decreased time to first antibiotic intervention, time to most targeted antibiotics, and antibiotic intensity score at 96 h after positive blood culture. This is essential in improving antimicrobial stewardship programs and minimizing unintended consequences of antibiotic use across hospital systems. Pharmacists can play a crucial role in interpreting AST results, identifying ineffective therapy, and contacting attending providers to suggest escalation, de-escalation, or other modifications to therapeutic regimens. In addition, hospital LOS for patients in the post-AXDX implementation group was significantly shorter, which can have a substantial impact on decreasing hospital costs. Multicenter prospective studies are required to evaluate the impact of fast ID and AST implementation via AXDX and its effects on clinical outcomes and antimicrobial stewardship programs, but the value of its use in this study is undeniable.

## MATERIALS AND METHODS

### Study design and antimicrobial stewardship protocol.

A pre-post quasiexperimental study of 200 patients (100 for pre-AXDX implementation and 100 for post-AXDX implementation) was conducted at Peninsula Regional Medical Center (PRMC), a 288-bed community hospital in Salisbury, MD. PRMC has 24 ICU beds, utilizes the EPIC electronic medical record system, and is a level III trauma center. We chose 100 patients for each group after reviewing GNR and fungal bacteremia occurrence rates at our institution. Due to lower anticipated numbers in comparison to those of other tertiary centers, we determined that targeting 100 patients in each group was pragmatic and comparable to published literature on rapid testing (7–12). All patients with blood cultures positive for Gram-negative rods (GNRs) or yeast observed on Gram stain and hospital admission for >24 h were evaluated for inclusion. Patients with a prior positive blood culture(s) within the past 7 days or who were deceased, on comfort care or hospice status, or designated for organ donation at the time of the positive blood culture were excluded from the study. Data collected included patient age, sex, level of immunosuppression, diagnosis of septic shock, Charlson comorbidity score, prior hospitalization within 90 days of blood culture draw, hospital length of stay (LOS), intensive care unit (ICU) days, 30-day readmission from blood culture draw, antibiotic therapy administered, infection source, and other clinical variables (17). The Peninsula Regional Medical Center institutional review board approved this study protocol.

### Standard-of-care microbiology workflow prior to implementation of AXDX.

VERIGENE system testing for GNR ID followed by MicroScan WalkAway system (Beckman Coulter, Inc., Brea, CA) for final AST was the standard of care in the pre-AXDX implementation group. The pre-AXDX study period included 100 patients from January 2017 to August 2017. Off-panel pathogen IDs were performed on MicroScan.

### Microbiology workflow with implementation of AXDX.

Implementation of AXDX at PRMC occurred on 4 December 2017. The post-AXDX implementation group consisted of fast ID and AST with the Accelerate Pheno system and Accelerate PhenoTest BC kit (Accelerate Diagnostics, Inc., Tucson, AZ) for positive blood cultures with Gram-negative rods or yeast observed on Gram stain. The post-AXDX study group included 100 patients from May 2018 to December 2018. Off-panel pathogen IDs were performed on MicroScan.

### Microbiology laboratory reporting and antimicrobial stewardship interventions.

**FIG 2:**
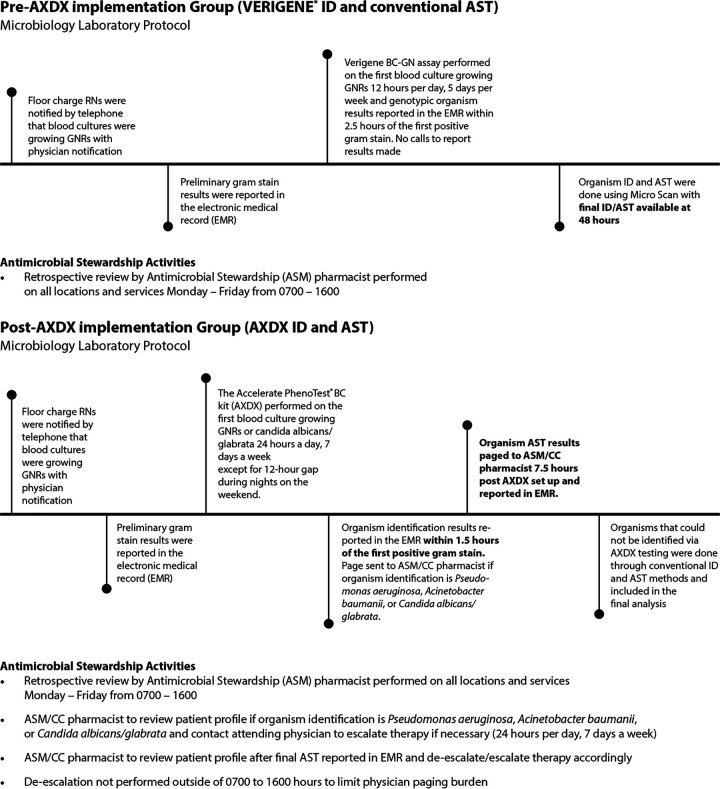
Comparison of laboratory protocol and antimicrobial stewardship activities.

Microbiology laboratory protocol and antimicrobial stewardship interventions for pre-AXDX and post-AXDX implementation groups are summarized in Fig. 2. All other aspects of pharmacy antimicrobial stewardship services remained unchanged.

### Measured endpoints and clinical assessment.

The primary endpoints measured were time to first antibiotic intervention, time to most targeted antibiotic therapy, and 14-day in-hospital mortality. Secondary endpoints included hospital and intensive care unit (ICU) length of stay (LOS), antibiotic intensity score at 96 h, and 30-day readmission rates.

Time to first antibiotic intervention was defined as the time from initial antibiotic(s) order to initiation, escalation, de-escalation, or discontinuation of one or more antibiotics, or switch to an antibiotic regimen with a higher or lower antibiotic intensity score (Table 3). Most targeted antibiotic therapies were defined as the narrowest antibiotic regimen acceptable for the source of infection in addition to the isolated organism’s susceptibilities. Antibiotic intensity score, developed internally, was calculated as the total score of all antibiotics administered at 96 h and used as a scoring system to measure antimicrobial de-escalation as described in the literature (18, 19).

**TABLE 3:** Antimicrobial rank system used for antibiotic intensity scoring (at 96 h of therapy)

Antimicrobial or antifungal	Rank (score)
Antimicrobial	
Antipseudomonal carbapenems	5
Antipseudomonal penicillin-penicillinase combinations, aztreonam, ceftazidime, ertapenem	4
Aminoglycosides, intravenous fluoroquinolones	3
Amoxicillin-clavulanic acid, ampicillin-sulbactam, 2nd-generation cephalosporins, 3rd-generation cephalosporins (except ceftazidime), oral fluoroquinolones, tetracyclines, trimethoprim-sulfamethoxazole, daptomycin, linezolid, vancomycin	2
Amoxicillin, ampicillin, 1st-generation cephalosporins, clindamycin, macrolides, metronidazole, nafcillin, penicillin, rifampin	1
None	0
Antifungal	
Amphotericin B	3
Micafungin	2
Fluconazole	1
None	0

### Statistical analysis.

For comparison of the categorical variables between the two groups, Fisher exact tests or chi-square tests were used as appropriate. Fourteen-day mortality was compared using Fisher’s test. The Wilcoxon rank sum test was used for the comparison of continuous variables such as average antibiotic intensity score, antibiotic days of broad-spectrum therapy (defined as initial empirical antimicrobial therapy), hospital LOS, ICU LOS, time to first antibiotic intervention, and time to most targeted antibiotics. JMP 13.0.0 software (SAS Institute Inc., Cary, NC) was used to perform statistical analyses. All tests were two-tailed, and a *P* value of <0.05 was deemed statistically significant.

## Supplementary Material

Supplemental file 1

## References

[1] Suárez CJ, Lolans K, Villegas MV, Quinn JP 2005 Mechanisms of resistance to beta-lactams in some common Gram-negative bacteria causing nosocomial infections. Expert Rev Anti Infect Ther 3:915–922. doi:10.1586/14787210.3.6.915. 16307504

[2] Ibrahim EH, Sherman G, Ward S, Fraser VJ, Kollef MH 2000 The influence of inadequate antimicrobial treatment of bloodstream infections on patient outcomes in the ICU setting. Chest 118:146–155. doi:10.1378/chest.118.1.146. 10893372

[3] Kang CI, Kim SH, Park WB, Lee KD, Kim HB, Kim EC, Oh MD, Choe KW 2005 Bloodstream infections caused by antibiotic-resistant Gram-negative bacilli: risk factors for mortality and impact of inappropriate initial antimicrobial therapy on outcome. Antimicrob Agents Chemother 49:760–766. doi:10.1128/AAC.49.2.760-766.2005. 15673761 PMC547233

[4] Singer M, Deutschman CS, Seymour CW, Shankar-Hari M, Annane D, Bauer M, Bellomo R, Bernard GR, Chiche JD, Coopersmith CM, Hotchkiss RS, Levy MM, Marshall JC, Martin GS, Opal SM, Rubenfeld RS, Poll TV, Vincent JL, Angus DC 2016 The third international consensus definitions for sepsis and septic shock (Sepsis-3). JAMA 315:801–810. doi:10.1001/jama.2016.0287. 26903338 PMC4968574

[5] Peker N, Couto N, Sinha B, Rossen JW 2018 Diagnosis of bloodstream infections from positive blood cultures and directly from blood samples: recent developments in molecular approaches. Clin Microbiol Infect 24:944–955. doi:10.1016/j.cmi.2018.05.007. 29787889

[6] Maurer FP, Christner M, Hentschke M, Rohde H 2017 Advances in rapid identification and susceptibility testing of bacteria in the clinical microbiology laboratory: implications for patient care and antimicrobial stewardship programs. Infect Dis Rep 9:6839. doi:10.4081/idr.2017.6839. 28458798 PMC5391540

[7] Avdic E, Wang R, Li DX, Tamma PD, Shulder SE, Carroll KC, Cosgrove SE 2017 Sustained impact of a rapid microarray-based assay with antimicrobial stewardship interventions on optimizing therapy in patients with Gram-positive bacteraemia. J Antimicrob Chemother 72:3191–3198. doi:10.1093/jac/dkx267. 28961942

[8] Heyerly A, Jones R, Bokhart G, Shoaff M, Fisher D 2016 Implementation of a pharmacist-directed antimicrobial stewardship protocol utilizing rapid diagnostic testing. Hosp Pharm 51:815–822. doi:10.1310/hpj5110-815. 27928186 PMC5135429

[9] Banerjee R, Teng CB, Cunningham SA, Ihde SM, Steckelberg JM, Moriarty JP, Shah ND, Mandrekar JN, Patel R 2015 Randomized trial of rapid multiplex polymerase chain reaction-based blood culture identification and susceptibility testing. Clin Infect Dis 61:1071–1080. doi:10.1093/cid/civ447. 26197846 PMC4560903

[10] Lockwood AM, Perez KK, Musick WL, Ikwuagwu JO, Attia E, Fasoranti OO, Cernoch PL, Olsen RJ, Musser J 2016 Integrating rapid diagnostics and antimicrobial stewardship in two community hospitals improved process measures and antibiotic adjustment time. Infect Control Hosp Epidemiol 37:425–432. doi:10.1017/ice.2015.313. 26738993

[11] Box MJ, Sullivan EL, Ortwine KN, Parmenter MA, Quigley MM, Aguilar-Higgins LM, MacIntosh CL, Goerke KF, Lim RA 2015 Outcomes of rapid identification for gram-positive bacteremia in combination with antibiotic stewardship at a community-based hospital system. Pharmacotherapy 35:269–276. doi:10.1002/phar.1557. 25809178

[12] Huang AM, Newton D, Kunapuli A, Gandhi TN, Washer LL, Isip J, Collins CD, Nagel JL 2013 Impact of rapid organism identification via matrix-assisted laser desorption/ionization time-of-flight combined with antimicrobial stewardship team intervention in adult patients with bacteremia and candidemia. Clin Infect Dis 57:1237–1245. doi:10.1093/cid/cit498. 23899684

[13] Marschal M, Bachmaier J, Autenrieth I, Oberhettinger P, Willmann M, Peter S 2017 Evaluation of the Accelerate Pheno System for fast identification and antimicrobial susceptibility testing from positive blood vultures in bloodstream infections caused by Gram-negative pathogens. J Clin Microbiol 55:2116–2126. doi:10.1128/JCM.00181-17. 28446572 PMC5483913

[14] Dare MK, McCain K, Lusardi K, Daniels K, Painter J, Lakkad M, Emery N, Rosenbaum E, Bariola JR 2018 Impact of Accelerate Pheno™ system rapid blood culture detection system on laboratory and clinical outcomes in bacteremic patients. IDWEEK2018, San Francisco, CA. https://idsa.confex.com/idsa/2018/webprogram/Paper70067.html.

[15] Ehren K, Meißner A, Jazmati N, Wille J, Jung N, Vehreschild JJ, Hellmich M, Seifret H 2020 Clinical impact of rapid species identification from positive blood cultures with same-day phenotypic antimicrobial susceptibility testing on the management and outcome of bloodstream infections. Clin Infect Dis 70:1285–1293. doi:10.1093/cid/ciz406. 31094414

[16] Seddon MM, Bookstaver PB, Justo JA, Kohn J, Rac H, Haggard E, Mediwala KN, Dash S, Al-Hasan MN 2019 Role of early de-escalation of antimicrobial therapy on risk of Clostridioides difficile infection following enterobacteriaceae bloodstream infections. Clin Infect Dis 69:414–420. doi:10.1093/cid/ciy863. 30312362

[17] Charlson ME, Pompei P, Ales KL, Mackenzie CR 1987 A new method of classifying prognostic comorbidity in longitudinal studies: development and validation. J Chronic Dis 40:373–383. doi:10.1016/0021-9681(87)90171-8. 3558716

[18] Madaras-Kelly K, Jones M, Remington R, Caplinger C, Huttner B, Samore M 2015 Description and validation of a spectrum score method to measure antimicrobial de-escalation in healthcare associated pneumonia from electronic medical records data. BMC Infect Dis 15:197. doi:10.1186/s12879-015-0933-9. 25927970 PMC4418054

[19] Madaras-Kelly K, Jones M, Remington R, Hill N, Huttner B, Samore M 2014 Development of an antibiotic spectrum score based on Veterans Affairs culture and susceptibility data for the purpose of measuring antibiotic de-escalation: a modified Delphi approach. Infect Control Hosp Epidemiol 35:1103–1113. doi:10.1086/677633. 25111918 PMC4778427

