# Supplementary material for: Pharmacist-Driven Implementation of Fast Identification and Antimicrobial Susceptibility Testing Improves Outcomes for Patients with Gram-Negative Bacteremia and Candidemia
Source: Antimicrob Agents Chemother. 2020 Aug 20;64(9):e00578-20. doi: 10.1128/AAC.00578-20 (PMC7449197; doi:10.1128/AAC.00578-20)
Supplement: Supplemental file 1 [file AAC.00578-20-s0001.pdf]

**Supplementary Table 1.** Final Organism ID of Positive Blood Cultures in pre-AXDX and post-AXDX Implementation Group

| **Pre-AXDX** | | **Post-AXDX** | |
|---|---|---|---|
| **Organism** | **N (%)** | **Organism** | **N (%)** |
| *Escherichia coli*[a] | 47 (56) | *Escherichia coli*[a] | 41 (46) |
| *Klebsiella pneumoniae* | 12 (14) | *Klebsiella* species | 17 (19) |
| *Proteus mirabilis* | 6 (7) | *Proteus* species | 6 (7) |
| *Escherichia coli* (ESBL) [a] | 5 (6) | *Enterobacter* species | 5 (6) |
| *Pseudomonas aeruginosa* | 4 (5) | *Pseudomonas aeruginosa* | 4 (4) |
| *Enterobacter cloacae* | 2 (2) | *Serratia marcescens* | 2 (2) |
| *Klebsiella pneumonia* (ESBL) | 2 (2) | *Bacteroides fragilis*[b] | 2 (2) |
| *Enterobacter aerogenes* | 2 (2) | *Citrobacter* species[b] | 2 (2) |
| *Klebsiella oxytoca* | 1 (1) | *Providencia stuartii*[b] | 1 (1) |
| *Proteus vulgaris* | 1 (1) | *Salmonella* species[b] | 1 (1) |
| *Serratia marcescens* | 1 (1) | *Moraxella osolensis*[b] | 1 (1) |
| *Candida glabrata* | 1 (1) | *Prevotella melaninogenica*[b] | 1 (1) |
| | | *Shingobacterium* species[b] | 1 (1) |
| | | *Vibrio vulnificus*[b] | 1 (1) |
| | | *Agrobacterium radiobacter*[b] | 1 (1) |
| | | *Candida albicans* | 1 (1) |
| | | *Stenotrophomonas* species[b] | 1 (1) |
| | | *Clostridium* species[b] | 1 (1) |

ESBL = extended-spectrum beta lactamase

[a] Total *E.coli* species significantly different between pre-AXDX and post-AXDX groups ($p = 0.036$)

[b] Organisms not identified by AXDX

**Supplementary Table 2.** Targeted Antibiotic Regimens in pre-AXDX and post-AXDX Implementation Group

| Pre-AXDX | | Post-AXDX | |
|---|---|---|---|
| **Antimicrobial Regimen** | **N (%)** | **Antimicrobial Regimen** | **N (%)** |
| Ceftriaxone | 55 (65) | Ceftriaxone | 52 (58) |
| Ciprofloxacin | 9 (11) | Ciprofloxacin | 10 (12) |
| Ertapenem | 5 (6) | Ceftazidime | 4 (4) |
| Piperacillin-tazobactam | 3 (4) | Ertapenem | 3 (3) |
| Imipenem-cilastatin | 3 (4) | Piperacillin-tazobactam | 4 (4) |
| Ceftriaxone + azithromycin | 2 (2) | Levofloxacin | 2 (2) |
| Cefdinir | 1 (1) | Cefazolin + fluconazole | 1 (1) |
| Ceftriaxone + metronidazole | 1 (1) | Ceftazidime + doxycycline | 1 (1) |
| Levofloxacin | 1 (1) | Ceftriaxone + azithromycin | 1 (1) |
| Micafungin | 1 (1) | Ceftriaxone + vancomycin | 1 (1) |
| N/A[a] | 3 (4) | Ciprofloxacin + vancomycin | 1 (1) |
| | | Ciprofloxacin + imipenem-cilastatin | 1 (1) |
| | | Ciprofloxacin + metronidazole | 1 (1) |
| | | N/A[a] | 7 (8) |

[a] Initial regimen continued for entire duration of therapy
